# Coexistant Meckel’s diverticulum and patent urachus: a case report

**DOI:** 10.1097/RC9.0000000000000253

**Published:** 2026-02-13

**Authors:** Cheikh Tidiane Mbaye, Florent Tshibwid A Zeng, Cheikh Seye, Christ Momo Tsague, Omar Sow, Cheikh Diouf

**Affiliations:** a Department of surgery and surgical specialties, Université Assane Seck, Ziguinchor, Senegal; b Unit of Pediatric Surgery, Ziguinchor Regional Hospital Center, Ziguinchor, Senegal; c Department of surgery, Faculty of Medicine, Université de Lubumbashi, Lubumbashi, Democratic Republic of the Congo; d Department of Pediatric Surgery, Diourbel Regional Hospital Center, Université Alioune Diop, Bambey, Senegal; e Department of visceral surgery, La Paix Hospital Center, Ziguinchor, Senegal

**Keywords:** coexistence, concurrent, Meckel’s diverticulum, neonate, patent urachus

## Abstract

**Introduction and Clinical Importance:**

Concurrent remnants of the omphalomesenteric duct and urachus are exceptionally found. Diagnosis of Meckel’s diverticulum is mainly incidental, but a patent urachus is usually clinically possible.

**Case Presentation:**

We admitted a 48-hour-old female patient for an eviscerated Meckel’s diverticulum with a history of urine smelling around the umbilicus. Preoperative ultrasound did not identify an associated urachal anomaly. During emergency surgical exploration, a patent urachus was identified. Both remnants were resected, with the Meckel’s diverticulum presenting gastric heterotopia. The postoperative course was unremarkable after a 2-year follow-up.

**Clinical discussion:**

An asymptomatic Meckel’s diverticulum is usually an incidental finding. In this patient, umbilical cord rupture due to a patent urachus eased the diagnosis. As ultrasound is operator dependent, its negation for associated urachal remnant must still indicate meticulous intraoperative exploration to definitively rule out concurrent urachal remnant when its association with an omphalomesenteric duct remnant is suspected.

**Conclusion:**

Association between Meckel’s diverticulum and patent urachus is rare, but possible. Identification of both anomalies is crucial for comprehensive management.

## Introduction

Meckel’s diverticulum (MD) is one of the most common congenital malformations of the gastrointestinal tract (GIT). It is a remnant of the omphalomesenteric duct (OMD), which establishes a communication between the midgut and the yolk sac. The latter provides nutrients in early pregnancy and regresses as the placenta gains in development. By the sixth to ninth week of intrauterine development, the OMD obliterates^[^1,2^]^. Failure of this process gives rise to remnants of the OMD, of which MD is the most common^[^2^]^. During the first four months of development, the allantois serves to empty the fetal bladder by establishing a communication between the bladder and the amniotic cavity^[^3^]^. The allantois subsequently undergoes obliteration to leave a fibrous remnant, which will form the median umbilical ligament (urachus)^[^3^]^. Patent urachus (PU) results from the complete failure of the obliteration process, leaving a communication between the bladder and the umbilicus^[^3^]^.

**HIGHLIGHTS**

- What is known on the subject: coexistence of remnants of the omphalomesenteric duct and urachus is very rare. The most common type is an association of patent omphalomesenteric duct and patent urachus.
- What this study adds: Rare coexistence of Meckel’s diverticulum and patent urachus in neonates. Preoperative diagnosis may be missed; careful intraoperative exploration is essential. Surgical excision of both remnants results in excellent outcomes.

**Figure 1.**
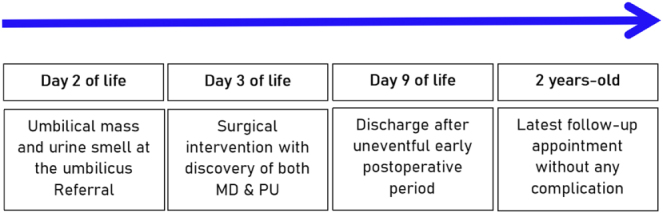
Timeline. The patient was received on day 2 of life and was surgically managed some hours later, with unremarkable postoperative period till the last follow-up at 2 years of age.

When MD is found in 2% of the population, PU is rarer, occurring in 1–2/100 000 deliveries^[^2,4^]^. However, the association between an OMD and urachal remnants is exceptionally reported in the literature^[^5^]^. We describe an exceptional case of a neonate with MD and PU managed at our department (Timeline summarized in Fig. 1). This case report has been written in line with the SCARE 2025 checklist^[^6^]^.

## Case presentation

A 48-hour-old female neonate was referred for an umbilical mass with a urine smell, identified during the initial postnatal examination. She was full-term born to a 31-year-old mother, gravida 2, para 2. Pregnancy was monitored with four prenatal visits and a single second-trimester ultrasound, which had normal results. Delivery was vaginal, without the need for perinatal resuscitation. The birth weight was 3100 grams.

**Figure 2.**
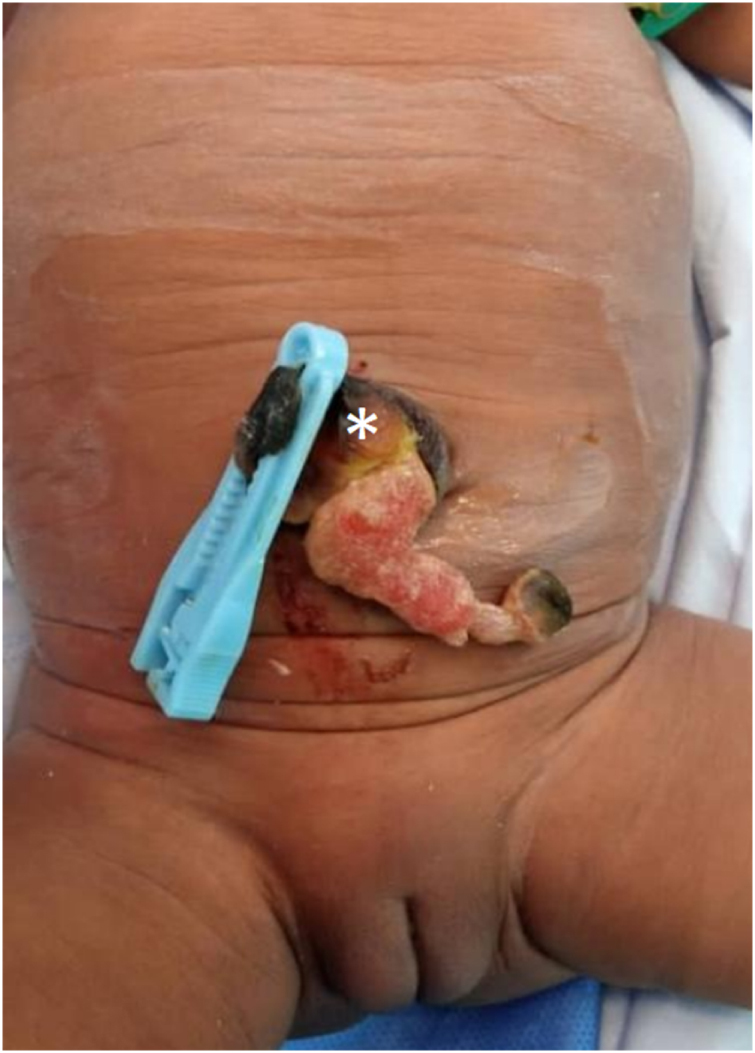
Clinical appearance. Ruptured umbilical sac (white asterisk) with protruding blind mass, without any leakage.

Upon admission, the general condition was good, with normal neurological behavior and vital signs within normal ranges. Inspection of the umbilical region revealed a desiccated, ruptured umbilical cord sac, a pinkish, 3-cm blind structure protruding through the umbilical ring. No other anomaly was noticed at this stage (Fig. 2). Abdominal ultrasound to look for an associated urachal anomaly (since the umbilicus was urine-smelling) did not find any anomaly. Diagnosis of ruptured umbilical cord with eviscerated MD was retained. An exploratory laparotomy was indicated, and the patient received intravenous (IV) antibiotics, analgesics, and hydration.

**Figure 3.**
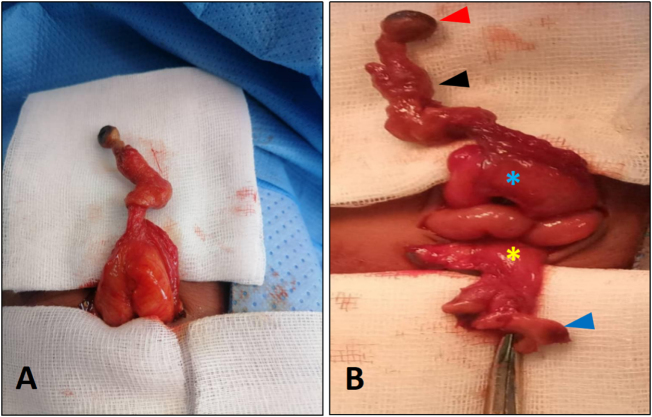
Intraoperative findings. (A) Identification of the MD and its ileal implantation. (B) The MD (black arrow) was attached to the umbilical skin (red arrow) and implanted on the ileum (blue asterisk). A PU was found (blue arrow) originating from the bladder (yellow asterisk). MD, Meckel’s diverticulum; PU, patent urachus.

The surgical intervention was carried by a senior pediatric surgeon resident in the fifth and last year of pediatric surgery residency, without previous experience of such a combination of MD and PU. Through a trans-umbilical approach, the ruptured umbilical cord was removed, and dissection of the fascia and peritoneum was carried out and allowing exposition of the ileum. The eviscerated structure had features of a fixed MD, located at 35 cm from the ileocecal junction (Fig. 3A). When attempting to ligate the urachus, urine was seen coming through it. After careful dissection, the urachus was fully connected to the bladder, with complete patency, allowing urine to be discharged at the umbilicus. This correlated with parents’ reporting urinary smell at the umbilicus. The intraoperative diagnosis was coexistent MD and PU (Fig. 3B). For MD, intestinal resection was performed, removing the MD with 2 cm margins on each side, followed by an end-to-end anastomosis. The PU was excised, with removal of its implantation to the bladder. The umbilical ring was closed with interrupted sutures with Polyglactin 2/0, and the skin was closed with a purse string using Polyglactin 5/0.

In the postoperative period, IV paracetamol was given for 5 days. Stools were passed 27 hours after surgery, so enteral feeding was immediately commenced and well tolerated by 36 hours after surgery, and the child was subsequently discharged 6 days postoperatively. The histopathological examination of the MD confirmed its nature as a true diverticulum, with gastric heterotopia (oxyntic glands) at its tip. The margins of resection were free of any gastric tissue. Microscopy of the resected PU showed its lining by a columnar epithelium, whereas its bladder implantation was lined by transitional epithelium. The patient underwent regular follow-up and, at 2 years postoperatively, remained asymptomatic.

## Discussion

We reported the case of a neonate with concurrent MD and PU, with the latter being missed on preoperative ultrasound and only intraoperatively diagnosed. Both anomalies were successfully managed with a good postoperative outcome.

MD and PU both result from faulty regression of the OMD and the allantois, respectively. The concurrence of remnants of OMD and the allantois in patients has been rarely reported in the literature^[^2–5^]^. A multitude of combinations between the main remnants of the OMD and those of the urachus can happen. In the literature, 50% of reported combinations in the pediatric population are patent OMD and PU^[^4–8^]^. The association between MD and PU, such as in our patient, is one of the rarest^[^4^]^.

Patients’ age at presentation depends on the symptoms of the remnants. Patients with patent OMD or PU tend to present during the neonatal period^[^5^]^. This may be linked to careful neonatal examination, as well as early symptoms with a moist umbilicus or discharge through the umbilicus. Patients with a combination of MD and a urachal remnant tend to present beyond the first year, mainly with a feature of complication related to MD, such as intestinal obstruction^[^7,8^]^. In a case similar to ours (PU and MD), the patient presented on day 10 of life, with a moist umbilicus^[^4^]^. In our case, we presume that urine leaking through the umbilicus accelerated desiccation of the umbilical cord, with its subsequent rupture, causing evisceration of the MD. This, combined with the urine smell at the umbilicus, led to the earlier patient’s referral. Urine smell at the umbilicus must alert the clinician to a possible PU. When the latter is diagnosed, one must formally rule out concurrent MD with imaging or careful surgical exploration. Inversely, diagnosis of MD must warrant research of associated PU.

**Figure 4.**
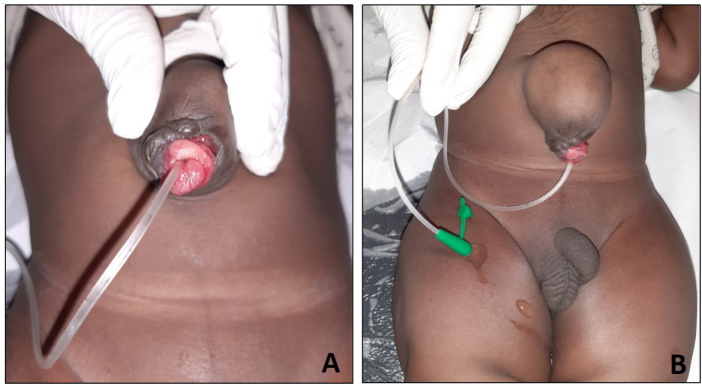
Clinical confirmation of patent urachus in a patient with an umbilical hernia. (A) The umbilical orifice of PU is catheterized. Note the presence of granulation tissue. (B) A clear liquid (urine) is leaking through the trans-umbilical catheter. In this patient, posterior urethral valves were diagnosed later on. MD, Meckel’s diverticulum; PU, patent urachus.

Diagnosis of MD is mainly incidental, except for symptomatic ones, with patients presenting with digestive hemorrhage, intestinal obstruction, diverticulitis, or, at adult age, malignant transformation^[^9,10^]^. Diagnosis of PU is usually straightforward, with history and clinical findings of spontaneous urine leakage at the umbilicus or by catheterization of the umbilical orifice of the PU, as demonstrated in another patient managed by the authors (Fig. 4). However, due to the presence of a granuloma, urine leakage may not be evident. In such cases, abdominal ultrasound is helpful^[^4^]^. But in our case, no anomaly was found, probably due to the lack of experience of the operator. In fact, with an experienced ultrasound operator, authors have reported preoperative diagnosis of MD associated with PU^[^4^]^. Such cases of combination of OMD and urachus remnants are a strong reminder of careful preoperative imaging, which is essential to lead the surgical indication.

Management of such a combination requires ablation of both remnants. The rationale of MD resection in children is due to the higher risk of it being symptomatic with age and due to the higher frequency of ectopic tissue, which may be gastric or pancreatic^[^9^]^. In our patient, a gastric ectopic tissue was found at the tip of the MD. This kind of ectopic tissue is known for generating hemorrhagic complications of MD^[^1,9^]^. Ablation of PU is justified by its risk of malignant transformation within adenocarcinoma, mainly found in adults^[^4^]^. Surgical techniques of MD management include three possibilities: diverticulectomy, wedge resection and segmental resection of the bearing loop^[^9^]^. In a similar case, diverticulectomy was performed and resulted in postoperative anastomotic stenosis, requiring re-operation^[^4^]^. In our patient, with segmental resection of the bearing loop and end-to-end anastomosis, no complication occurred.

### Strengths and limitations

This study reports an extremely rare association between a MD and patent urachus. Its main limitation is the missed preoperative diagnosis of associated patent urachus, both clinically and on ultrasound.

### Patient perspective

The patient’s parents were happy with the successful repair of both anomalies and disappearance of the urine smell at the umbilicus.

## Conclusion

Association between remnants of the OMD and those of the urachus is extremely rare, but possible. Urine smell at the umbilicus must make the clinician suspect a patent urachus, even in the absence of visible urine dribbling at the umbilicus. Before surgical exploration, imaging investigation is essential to avoid intraoperative surprise and plan for comprehensive management of both remnants. Surgical exploration, guided by parents’ complaints (urine smelling at the umbilicus), is helpful to identify preoperatively missed patent urachus. Resection of both remnants provides a good long-term outcome.

## Data Availability

The data that support the findings of this study are available from the corresponding author upon reasonable request.
